# Impact of Inflammatory Bowel Disease upon Growth in Children and Adolescents

**DOI:** 10.5402/2011/365712

**Published:** 2011-04-05

**Authors:** V. Moeeni, A. S. Day

**Affiliations:** ^1^Department of Paediatrics, University of Otago, Christchurch, Riccarton Avenue, Christchurch 8140, New Zealand; ^2^Department of Paediatrics, Christchurch Hospital, Christchurch 8140, New Zealand

## Abstract

The inflammatory bowel diseases (IBDs) are chronic inflammatory processes affecting the gastrointestinal tract. When diagnosed in childhood and adolescence, IBD almost always impacts adversely upon the nutritional state of the patient. Weight loss and impaired linear growth may be present at diagnosis or subsequently. Further potential nutritional consequences in childhood IBD include malnutrition, anaemia, osteopaenia, and delayed puberty. Understanding the nutritional aspects of IBD is paramount in growing children, especially those entering and advancing through puberty. This paper focuses upon the nutritional impacts of IBD in children and adolescents.

## 1. Introduction

The inflammatory bowel diseases (IBDs) are chronic illnesses affecting the gastrointestinal tract: they predominantly comprise Crohn's disease (CD) and ulcerative colitis (UC). Although CD and UC share some features, they have distinctive endoscopic and histological characteristics and disease patterns [1]. Furthermore, the clinical manifestations and outcomes of these two conditions vary. At present, there are no specific medical cures for CD and UC: current therapies aim to control the disease and prevent adverse outcomes [1]. 

CD and UC may present at any age. Although IBD is uncommon in the first decade of life, presentation in infancy is well recognized. IBD is more common in the second decade, particularly in adolescence [2, 3]. There are clear demonstrations of increasing incidence of IBD around the world over the last decades, with increased rates also noted in areas with previous very low rates [3–8]. The changing incidence of IBD has also been noted in paediatric populations, with particular increases in CD [9, 10]. 

A history of weight loss or poor weight gain is commonly recorded at the time of diagnosis with IBD in children and adolescents. Poor linear growth may also be evident at the initial assessment, leading to growth failure in some. Growth impairment is typically more prominent in CD than UC [1]. Impaired nutrition may lead onto short- and long-term consequences that include delayed puberty, micronutrient deficiencies, impaired adult height, and bone disease. Regular monitoring and detailed attention to nutritional support remains a central facet of the management of children and adolescents with IBD. This paper will focus on the impact of IBD upon the nutritional state of children and adolescents and will highlight recent advances in this area.

## 2. Altered Growth Patterns in Paediatric IBD

### 2.1. Weight in Children with IBD

Numerous cohort studies have demonstrated weight loss or poor weight gains at the time of initial diagnosis of IBD. Reports indicate that most children with CD have a history of weight loss or absent weight gains prior to diagnosis. In a cohort of 386 Canadian children diagnosed with CD over a ten-year period, 80% had a history of weight loss [1]. A retrospective chart review of a cohort of 61 children diagnosed with CD at Sydney Children's Hospital, Sydney, Australia showed that 51% had a history of weight loss at the time of diagnosis [11]. In a prospective UK study of 379 paediatric CD patients, 58% had weight loss at diagnosis and 27% had weight < 3rd centile [12]. Weight loss tends to be seen less commonly in children with UC—up to 31% of 172 children in the same UK cohort had a history of weight loss at diagnosis [12].

Maintaining appropriate weight gain also continues to be an ongoing issue after diagnosis. In a cohort of 41 children with longstanding CD recruited to assess dietary intakes and nutrition, the 18 children with active CD had lower mean *Z* scores for weight, height, and BMI than the 23 children currently in remission [13].

The reasons for altered weight gains are likely multifactorial, with decreased caloric intake being the most common [14–19]. Poor intake may be the consequence of the anorexic effects of proinflammatory mediators, such as interleukin-1*β* or Tumour necrosis factor (TNF)-*α* [20, 21] or result from early satiety, pain, or nausea. Pain associated with eating, leading to poor tolerance of foods, and the fear of diarrhoea following meals are additional reported reasons for reduced intake. Small bowel involvement may lead to disaccharide intolerance resulting in shorter gut transit times, pain, and exacerbation of diarrhoea. Malabsorption of food components and the diversion of calories to sites of gut inflammation may be further contributing factors leading to impaired growth.

### 2.2. Impaired Linear Growth in IBD

In addition to presentation with loss of body weight or poor weight gains, children also commonly have altered patterns of linear growth. Decreased height velocity was noted in up to 88% of a group of 50 children at the time of diagnosis with CD—a number of these children had impaired linear growth prior to the onset of gut symptoms [22]. Impaired linear growth tends to be more pronounced in boys than girls—this is principally related to the male pubertal growth spurt occurring later and lasting longer. Boys may also be more aware of variations in height. Furthermore, the limited duration of puberty limits the time before impaired linear growth becomes irreversible [23, 24]. 

CD is commonly associated with impaired linear growth either at diagnosis or as a long-term outcome [22–29]. Some earlier studies reported that despite growth retardation occurring during teenage years, many of these patients eventually achieved heights within the normal range for the general population [30, 31]; however, the genetic component of linear growth was not considered in these studies. Markowitz et al. [24] retrospectively assessed the growth patterns of 48 adults with IBD (38 with CD and 10 with UC: 73% male) who had previously been diagnosed during childhood. These investigators used two height prediction methods and employed the American National Centre for Health Statistics growth charts: 31% of this group were found to have permanently impaired linear growth. In their study of 132 children and young adults aged between 5–25 years, Sentongo and colleagues [28] argued that height adjusted for genetic potential was a more powerful way of assessing height status in children with CD. In these children, the *Z* scores based on adjusted heights were significantly lower than *Z* scores based on measured heights. Again, males in this group were most severely affected, with the average adjusted height for age *Z* score one standard deviation less than expected. 

Lee et al. [30] recently undertook a prospective assessment of height in 295 children with CD and UC who had been diagnosed between 2002 and 2008 in Boston, USA. Twenty-two percent of these children (90% of whom had CD) had linear growth impairment (height for age *Z*-score of −1.64 or less) documented on one or more measurements from the time of diagnosis. The final mean adult height *Z* score in this cohort was −0.39.The main predictors of final adult height in this cohort were found to be lower parental height and the patient's lowest height *Z* score. 

A recent British study also assessed the final adult height of a group of 23 individuals who had been diagnosed with CD prior to their 16th birthday [31]. This group had a final adult height *Z* score of −0.29: very similar to that observed in the North American study [30]. The mean final height of these patients was 2.4 cm less than the parental or target height (range of −20.0 to +9.0 cm). Twenty percent of the group had a final adult height greater than 8.0 cm below their target height. Jejunal disease and the length of symptoms prior to diagnosis (diagnostic delay) were predictive factors of final adult height in this cohort. In this cohort, exposure to corticosteroids, surgical intervention, and parental height were not predictive factors. Jejunal disease location has previously been suggested to be associated with linear growth impairment [32, 33]. 

A Danish population-based assessment of growth in children with IBD highlights the differential effects of CD and UC upon growth [34]. Forty-four children with CD were included in this study, along with 50 with UC and four with IBD unclassified (IBDU). The growth data was normalised using Danish reference values. The children with UC had similar height and BMI scores for age to control data. In contrast, the children with CD had height for age *Z* score of −0.77: also they were shorter than the children with UC (*P* = .005). Fifty percent of the children with CD had a height less than the 25th centile for age.

### 2.3. Factors Influencing Linear Growth in Children with IBD

Numerous factors impact upon impaired linear growth in IBD. Several gut-derived mediators likely impact negatively upon linear growth [35]. TNF-*α* inhibits chondrocyte activity in growth plates [36]. Interleukin (IL)-6 may have direct effects leading to growth failure [37]. Elegant studies in rodent models show that approximately 40% of growth impairment is consequent to the effects of inflammatory proteins, especially IL-6 [38, 39]. Further, TNF-*α* and IL-6 both independently suppress levels of Insulin-like Growth Factor (IGF)-1, a critical mediator of the local actions of growth hormone [40].

Male gender, age at diagnosis, disease location, disease severity, and treatment modality may also all affect final height acquisition. Severity of disease (defined by various measures such as steroid requirements, use of immunosuppressives, and cumulative period of hospitalisation) was correlated with impaired weight and height gains in a cohort of Israeli children [41]. Location of disease in these children was also weakly associated with growth impairment. Interestingly, the presence of the NOD2 genotype was not associated with impaired weight or linear growth, despite associations between ileal location and mutations in this gene.

A recent report highlighted the importance of genetic influences upon linear growth in children with IBD. In a cross-sectional study, 951 subjects, comprising 317 CD patient-parent trios, were genotyped for growth-associated genetic loci and CD-associated loci [42]. Linear growth impairment in the children with CD was associated with a polymorphism in the dymeclin gene *DYM *(Odds Ratio 3.2).

### 2.4. Pubertal Delay in IBD

Delayed puberty is also frequently observed in children with IBD, especially those with CD [43, 44]. In one cohort of children diagnosed with CD prior to the onset of puberty, menarche was noted to occur at or later than 16 years of age in almost three-quarters of the girls [43]. Delayed pubertal development is more common in children who have never achieved remission or who have had repeated disease relapses [44], emphasizing the interactions between disease control and growth in children.

### 2.5. Overweight and Obesity in IBD

Although the most concern in regard to the nutritional impact of IBD in children is towards under nutrition, some children with IBD may be overweight (BMI > 85%) and/or obese (BMI > 95%). In a cohort of 166 children with newly diagnosed IBD from Wisconsin, USA, 12.1% of those with CD and 17.6% of those with UC were overweight/obese [45]. 

In a group of 1598 American children with known IBD, the prevalence of overweight and obese status was found to be 23.3% overall [46]. A higher rate (30%) was also seen in the UC group. Overweight/obesity in this multicentre cohort was associated with African-American ethnicity and Medicaid insurance status. In addition, a prior surgical intervention was linked with overweight/obese status in the children with CD, suggesting that the presence of over nutrition may be associated with a more severe disease course.

### 2.6. Influence of Medical Therapies upon Growth in Children with IBD

Medical therapies may impact negatively upon aspects of growth in children with IBD. Corticosteroids, for example, can lead to numerous side effects, including nutritional consequences [47]. Increased appetite and fluid retention are commonly seen after commencement of steroids. Consequently, apparent improvements in weight during a course of corticosteroids may reflect fluid retention and not an improvement in the underlying nutritional status. Steroids also lead to enhanced bone resorption and decreased new bone formation, adversely affecting bone health [48, 49]. Further, daily corticosteroid therapy can suppress IGF-1 activity, contributing to inhibition of linear growth and impaired final height [50]. 

Some medical therapies may compromise micronutrient status. Sulphasalazine may lead to folate malabsorption; however, daily folate supplementation does not appear necessary [51]. Folate supplementation is required, however, when Methotrexate is used to avoid folate deficiency due to the inhibition of the conversion of folate to the active moiety tetrahydrofolate [52]. Cholestyramine, when required for bile-salt diarrhea, may impede absorption of vitamin B12. 

In contrast, some interventions, such as exclusive enteral nutrition (EEN) and biological therapies, have numerous positive nutritional effects. EEN is a specific nutritional therapy that involves the administration of a liquid formula as sole nutritional intake (with exclusion of normal diet) for up to 8 weeks [53]. This therapy is efficacious in active CD, with remission rates similar to those seen with corticosteroids [54]. EEN leads to positive improvements in weight and linear growth, normalization of IGF-1 levels, and stabilization of bone turnover [53]. EEN is also associated with improved vitamin D status compared to children treated with corticosteroids [55]. 

Biological therapies in children with CD also clearly impact positively upon growth [56–60]. Walters et al. [58] examined the growth patterns of 32 children treated with Infliximab (28 responders) for severe CD. These children had improved height velocity, and height centile increases after Infliximab, with timing prior to early puberty being important. Malik et al. [59] found that improvements in linear growth after Infliximab can be independent of pubertal status and reduction in corticosteroids. These effects of infliximab in children with CD may be due to improved inflammation with mucosal healing or due to direct effects upon growth hormone and IGF-1 signaling [60]. In a recent report, Crombé et al. [61] demonstrated that the height *Z* scores of a group of 41 French children responding to Infliximab improved from −0.57 ± 1.18 to −0.25 ± 0.99 (*P* = .04) over the period of followup. In contrast, those children not responding to this drug had no catch up growth.

### 2.7. General Management of Growth in Children with CD

Given the frequency and patterns of disturbed growth in children with IBD (and especially those with CD), the management of such children must include a clear and constant focus upon growth and nutrition. Resumption of normal growth patterns should be seen as an element of the success of therapy. 

Baseline assessment of nutritional status at the time of diagnosis should include detailed standard anthropometry, along with clear documentation of preceding growth patterns and familial growth patterns (especially parental heights). Furthermore, close monitoring of growth during followup must be considered a mandatory part of the multidisciplinary care of children and adolescents with IBD.

## 3. Conclusion

There is now plentiful evidence demonstrating the significant impact of IBD upon growth and nutrition in children. Recent studies have helped to demonstrate the importance of nutritional, inflammatory, and genetic factors in growth outcomes. Consequently, a key aspect of the ongoing management of children and adolescents with IBD should be the close and constant attention to growth.
